# Population genomics of parallel evolution in gene expression and gene sequence during ecological adaptation

**DOI:** 10.1038/s41598-018-33897-8

**Published:** 2018-11-01

**Authors:** María José Rivas, María Saura, Andrés Pérez-Figueroa, Marina Panova, Tomas Johansson, Carl André, Armando Caballero, Emilio Rolán-Alvarez, Kerstin Johannesson, Humberto Quesada

**Affiliations:** 1 Departamento de Bioquímica, Genética e Inmunología, Universidad de Vigo, 36310 Vigo, Spain; 2 Department of Marine Sciences, Tjärnö, University of Gothenburg, SE-452 96 Strömstad, Sweden; 3 Department of Biology, University of Lund, SE-223 62 Lund, Sweden

## Abstract

Natural selection often produces parallel phenotypic changes in response to a similar adaptive challenge. However, the extent to which parallel gene expression differences and genomic divergence underlie parallel phenotypic traits and whether they are decoupled or not remains largely unexplored. We performed a population genomic study of parallel ecological adaptation among replicate ecotype pairs of the rough periwinkle (*Littorina saxatilis*) at a regional geographical scale (NW Spain). We show that genomic changes underlying parallel phenotypic divergence followed a complex pattern of both repeatable differences and of differences unique to specific ecotype pairs, in which parallel changes in expression or sequence are restricted to a limited set of genes. Yet, the majority of divergent genes were divergent either for gene expression or coding sequence, but not for both simultaneously. Overall, our findings suggest that divergent selection significantly contributed to the process of parallel molecular differentiation among ecotype pairs, and that changes in expression and gene sequence underlying phenotypic divergence could, at least to a certain extent, be considered decoupled processes.

## Introduction

The importance of natural selection on population divergence and the genesis of new species remains poorly understood. One reason for this limited knowledge is the stochasticity linked to the somewhat unique history of each population and species, which can overwhelm the fingerprint of adaptive divergence^1^. Instances of repeated, parallel phenotypic evolution in response to similar environmental pressures provide strong evidence of evolution by natural selection, as genetic drift is unlikely to generate a concerted change in multiple, independent lineages^2,3^. However, the underlying genetic basis of this process is unclear. In particular, we know very little as to whether selection acts upon the same genetic machineries to generate repeated phenotypes, or if its action follows alternative genetic routes^4–6^. Similarly, it remains unknown to what extent constraints faced by organic evolution might facilitate the repeated use of the same genes during independent phenotypic evolution^7,8^.

Evidence for parallel evolution has been found in many taxa^4,9^. However, one limitation of our view that parallel evolution is rather abundant comes from the fact that many studies are based on targeted candidate gene surveys that suffer from an inevitable ascertainment bias, as they do not allow answering whether repeated genetic changes are ubiquitous across the genome or more frequent than the neutral expectation^3^. Recent studies using a genome-wide approach have provided some unbiased insights into our understanding of the level of genome-wide repeatability linked to parallel evolution. For instance, molecular footprints of selection underlying parallel phenotypic evolution in cichlid fishes^10^, Australian groundsel^11^ and lake trout^12^ involve replicated evolution on a rather restricted subset of genes and, more frequently, divergence events that are unique to each population. Similarly, the early stages of parallel speciation in the stick insect *Tinema cristinae* involve mostly nonparallel divergence despite evidence of the importance of repeated selection on the same genes^13^. However, the repeatability of evolution through the reuse of the same genes may be substantial amongst recently diverged lineages^9,14^. Overall, these and other studies^15–18^ suggest that the genomic architecture underlying parallel phenotypic evolution frequently follows complex genetic trajectories, affecting multiple loci that show a mosaic pattern of both repeatable and idiosyncratic divergence, and where ancestral standing variation is frequently an important source of adaptive variation.

Very few studies have attempted to address the extent to which parallel gene expression differences and genomic divergence underlie parallel phenotypic traits^19–22^. This question is of central importance, because adaptive variation is likely to be underpinned by changes in both regulatory and coding sequences^23^. If evolution in coding sequences and regulatory regions are two highly related phenomena, then patterns of differentiation in coding sequence and gene expression should be markedly similar, i.e. they should be coupled. However, previous attempts to test the coupling between coding sequences and gene expression in multicellular organisms have given conflicting results, with markedly similar patterns of differentiation found in some datasets^24–27^, but very dissimilar in others^17,28,29^. To add further uncertainty, the specific mechanism underlying these observations remains elusive. Thus, adaptive selection driving rapid evolution of both gene expression and coding sequence may account for the coupling^24^, but also variation in functional constraints, in which genes less constrained in coding sequence would also be less constrained in expression^26–30^. Alternatively, markedly dissimilar patterns of differentiation would point towards the possibility that changes in coding sequence and gene expression underlying phenotypic evolution play different roles during evolution and could, at least to a certain extent, be considered decoupled processes^31,32^. Unveiling the degree of convergence at different levels of genomic organization will help to establish to what extent natural selection, genetic constraints, or independent modes of evolution, determine whether patterns of genetic differentiation associated with adaptation are predictable.

The marine snail *Littorina saxatilis* provides an excellent opportunity for testing these aspects of evolutionary repeatability. In this ovoviviparous species, pairs of ecotypes adapted to distinct parts of the same shores have repeatedly evolved in different geographical regions from Europe^33–35^. Parallel phenotypic divergence involves many traits, including body size, shell shape, shell thickness, and behavior^36^. Hybridization occurs in a relatively narrow zone, but gene flow among ecotypes is restricted due to assortative mating, immigrant inviability, and habitat choice^37–39^. Consistent with the prediction of parallel evolution, pairs of sympatric ecotypes cluster in phylogenetic trees by geographic origin but not by ecotype^40^. Computer simulations assessing the confounding effect of gene flow on phylogenetic inference confirm this result, demonstrating that the time elapsed since the emergence of ecotypes would not be enough to erode the distinctive phylogenetic signal linked to a parallel or a non-parallel (allopatric) origin of ecotypes^41^. Moreover, the comparison between alternative evolutionary models further supports that data better fit a scenario in which the separation of pairs of ecotypes occurred in parallel at both regional and local scales^35^. Recent genomic studies comparing populations from three geographically distant regions (Spain, Sweden and United Kingdom) suggest that footprints of selection are frequently region-specific^42,43^, or even site-specific at a very local scale^44^. Still, no study in *Littorina* has so far investigated the extent of parallelism in gene expression nor the relation between variation in gene expression and divergence in coding sequences.

Despite the ongoing development of next-generation sequencing technologies for genome-wide evolutionary analyses, it remains technically and financially unapproachable for many laboratories to sequence whole genomes or transcriptomes. Microarrays remain a simple and inexpensive alternative for genotype-related purposes and gene expression analyses^45^. Array-based comparative genomic hybridization can be accurately used as a proxy to estimate genome-wide divergence by comparing hybridization intensities of individuals on the microarray^46,47^. Modifications of this method have been successfully used to identify SNPs or copy number variants without the need of allele-specific probes, thanks to a linear relationship between hybridization signal and sequence divergence^47^. Similarly, microarrays remain widely used for gene expression profiling, as correlation between microarray data and other platforms such as RNA-seq is usually pretty good^48,49^. Although microarrays may be problematic for the study of low expressed genes, RNA-seq shows a greater degree of intensity-dependent variation than do microarrays^50^, which has led some authors to recommend the use of RNA-seq^51,52^, and others challenging that conclusion^53,54^.

Here we combine genome-wide evolutionary analyses of coding sequences and gene expression data using microarrays for investigating the molecular basis of adaptive divergence, employing *L. saxatilis* ecotypes from NW Spain as a model system. In this region, a large “crab ecotype” and a smaller “wave ecotype” have evolved repeatedly in response to crab predation and wave exposure respectively^33,35,40,55^. The recent origin of these ecotypes (<10,000 years)^35^ is expected to be associated with high levels of shared genetic constraints and standing variation that would facilitate a rapid and more pervasive repeated evolution. Our objectives were i) assess to what extent expression and sequence differences between ecotypes affect the same genes, ii) determine the level of correspondence between gene expression divergence and coding sequence divergence, and iii) quantify how natural selection may affect repeatability. Our results stress the important contribution that both gene regulation and coding regions can make to rapid phenotypic evolution and adaptation.

## Materials and Methods

### Sampling

**Figure 1:**
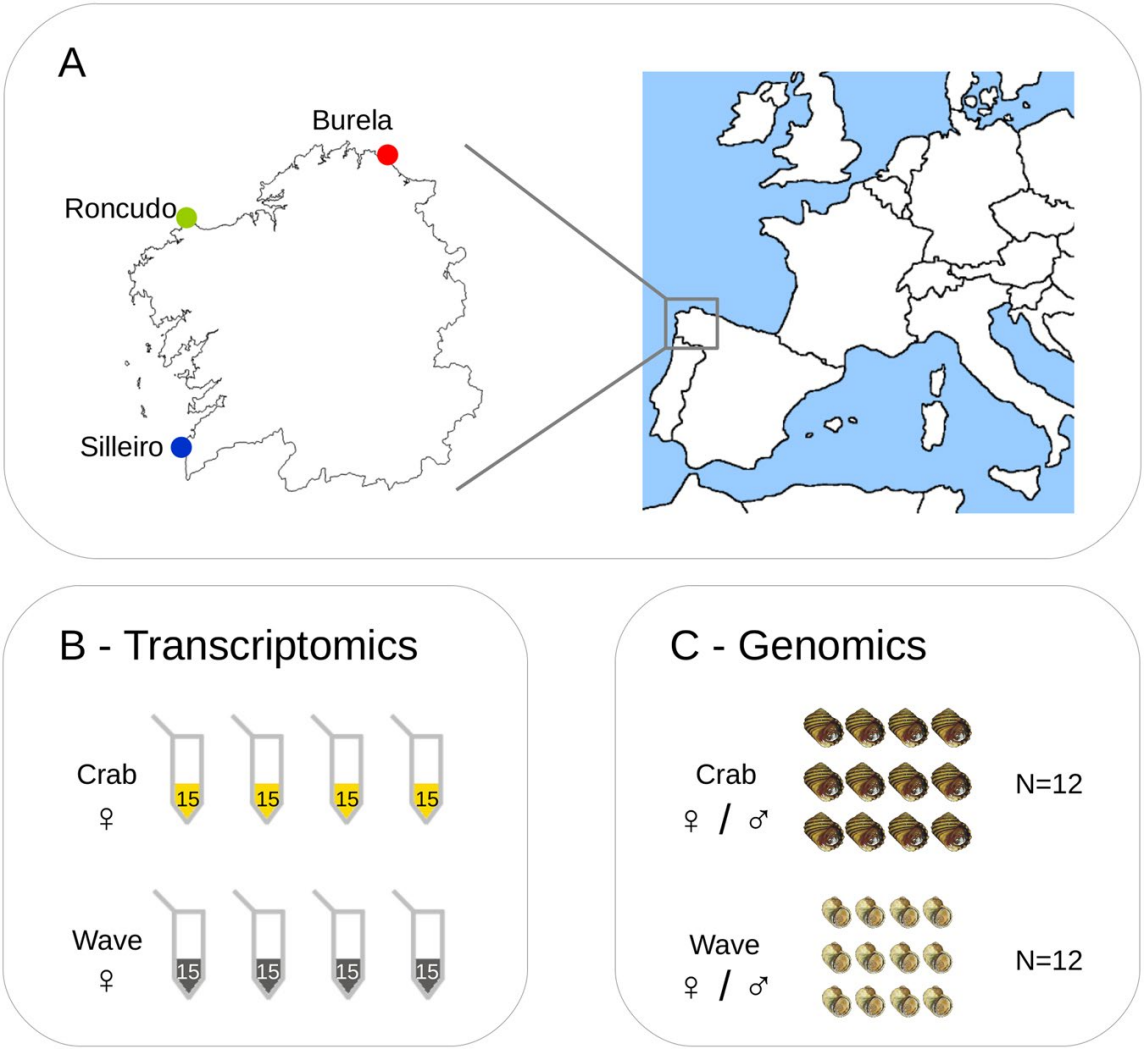
Experimental design. (**A**) Geographical situation of the three localities sampled in this study. (**B**) Transcriptomic scan: 4 pools were assayed for each ecotype and locality (24 pools in total); each pool consisted of 15 females with an equimolecular contribution to the pool. (**C**) Genomic sequence scan: 12 individuals were assayed for each ecotype and locality (72 individuals in total); each single individual was separately hybridized against the array.

Adult snails were collected in August 2010 from three Galician (NW Spain) localities: Burela (N 43°40′54.8′′; W 7°22′1.8′′), Roncudo (N 43°16.31′32′′; W 8°59.23′93′′′), and Silleiro (N 42°6′17.20′′; W 8°53′56.59′′) (Fig. 1). At each locality, specimens from the “crab” and the “wave” ecotypes were obtained from the upper and lower shore level respectively to avoid collecting intermediate forms (i.e. hybrids). Snails were immediately transported to our facilities in the ECIMAT Marine Station, and sexed according to the presence or absence of penis. Specimens used for DNA extractions were stored at −80 °C until processed. Specimens targeted for expression analysis were maintained alive in an aquarium under identical environmental conditions for two weeks using a continuous sea water flow (16 °C, 36.1‰ salinity, 6.8 mg/L oxygen level). This step aimed to minimize the impact of environmental variance on gene expression patterns by ensuring that all individuals shared the same environmental conditions prior to expression analysis. After this period, snails were stored at −20 °C in RNAlater solution (Ambion) until RNA extraction.

### RNA and DNA extraction

Total RNA was isolated from the foot muscle tissue of single females using TRIZOL reagent (Invitrogen) according to the manufacturer’s instructions. Females have the advantage of providing larger RNA yields than males given their bigger size, while displaying expression patterns similar to those from males across the different ontogenetic stages of each ecotype^56^. The Turbo DNA-freeTM kit (Ambion) was used to remove any remaining DNA from RNA extractions. Genomic DNA was isolated from the foot muscle tissue of single males and females using a CTAB extraction method^57^ modified to include RNAse treatment. DNA samples were further cleaned with NucleoSpin columns (Macherey-Nagel) following manufacturer’s instructions. RNA and DNA purity was assessed using a NanoDrop spectrophotometer (NanoDrop Tech. Inc., Wilmington, DE). All extractions were standardized to 100 ng/µL after checking their integrity in agarose gels.

### NimbleGen *L. saxatilis* microarray

The *L. saxatilis* oligonucleotide microarray^58^ was developed by NimbleGen Roche (090824_L_saxatilis_expr_HX12, 12 × 135K array format) on the basis of draft or versioned assemblies from the *Littorina saxatilis* EST database^59^ and the GenBank database. The microarray contained sequence information based on 25,205 partial transcripts, hereafter referred to as “genes” for simplicity, and which represent the coding part of the genome. These transcripts were obtained mainly by 454 sequencing of cDNA libraries from both the “crab” and “wave” ecotypes^59^. Each gene was usually represented on the array by five non-overlapping 60-nt probes. Each NimbleGen slide contained 12 identical subarrays. We used this microarray to assess variation in gene expression and also in genomic sequence using, for the latter, a comparative genomic hybridization (CGH) approach, which is based on hybridization of labeled DNA fragments to a microarray^46^. This allowed us to compare variation in expression and nucleotide sequence for the same subset of the *L. saxatilis* genome.

### Gene expression profiling

For this analysis, pools of total RNAs were retrotranscribed to cDNAs representing the coding part of the transcriptome, which were then compared to establish patterns of over- and under-expressed genes. We prepared four replicate samples from each ecotype and locality (24 samples in total), each including 15 pooled female specimens with an equimolar RNA contribution of each specimen to the pool. The RNA from each pool was retrotranscribed with the SuperScriptTM Double-Stranded cDNA Synthesis Kit (Invitrogen) following the conditions recommended by the manufacturer. Cy3 labeling was performed with the NimbleGen One-Color DNA labeling kit (Roche) using a starting amount of 1 µg of cDNA per pool. Then, for each pool, 4 µg of Cy3 labeled cDNA was resuspended in 12 µL of hybridization solution, of which 6 µL was applied onto a subarray. Pools were randomly distributed in the subarrays. Hybridization was carried out at 42 °C for 19 h on a NimbleGen Hybridization System with continuous mixing. After hybridization, the arrays were washed in buffers of various stringencies using the NimbleGen Washing kit. Arrays were scanned using an Agilent G2565AA microarray scanner (Agilent Technologies) with a resolution of 2 µm. The quality of the images was assessed using the NimbleScan v.2.5. software (NimbleGen/Roche), discarding those images with signal intensity or other metrics outside the range recommended by the manufacturer.

Data were extracted using NimbleScan v.2.5 and analyzed in the R/Bioconductor statistical environment. The pdfInfoBuilder and oligo^60^ packages were used for data handling and pre-processing, with the robust multichip average (RMA) method^61^ used for background correction, quantile normalization and probe-level summarization of the microarray samples. A single value was obtained for each gene, resulting from each summarization of probe-level data. To decide whether a gene was expressed, a threshold level representing the “background signal” was calculated based on the average hybridization signal of the empty spots present in the array. Genes for which more than 20% of the probes had an average hybridization signal lower than the “background signal” were disregarded^62^.

### Genomic divergence profiling

For the analysis of variation in genomic sequence, each subarray hosted the genomic DNA of one single individual and the genomic DNA of a common reference sample. Thus, in this experiment, genomic DNA was hybridized against the coding portion of the *L. saxatilis* genome represented in the microarray. The reference sample was composed of a DNA pool of 100 “crab” and “wave” snails from two British *L. saxatilis* locations (Dunvar and Thornwick, the latter used in the array design^58^), to ensure consistent and non-zero hybridization signals for the reference sample in all the probes from the array. Therefore, “crab” and “wave” Galician ecotypes should be equally diverged from both the array and the reference, as the latter included mostly (90%) individuals from the same location used in the array design.

The NimbleGen/Roche Dual label kit was used to label the reference sample (Cy5 dye) and the DNA of each specimen (Cy3 dye) following manufacturer´s instructions. Hybridization procedures, array scanning and quality control of the images obtained were performed as in the expression arrays, but increasing the amount of labeled DNA (20 µg) and incubation time (72 h). The whole experiment included 72 Galician snails (12 per ecotype and locality) for which genomic DNA extracts were individually hybridized to the array. The data from scanning pictures generated by NimbleScan were parsed using ringo^63^, an R/Bioconductor package. The signal intensity data for each channel was corrected for the local background signal using the normexp + offset method^64^, log_2_-transformed, and quantile normalized using the method proposed for two channels^65^, as implemented in the package limma for R/Bioconductor^66^. We performed a probe-level data analysis to test DNA sequence differences between the distinct gene fragments included in a probe set and the hybridized DNA.

Low hybridization signals (below 10.35) in the *L. saxatilis* microarray may correspond in some instances to probes spanning exon boundaries and/or untranslated regions^58^. To account for this possible source of noise in our data, and also to exclude probes that were not accurately detected in the array, we have filtered these sequences by removing probes with an average hybridization signal lower than the “background signal” (i.e. 10.7) of each channel. Untranslated regions would similarly generate low hybridization signals in the expression study, and these were also removed from the data (see above). Only genes containing probes that simultaneously passed genome and expression profiling filters were used in the subsequent analyses, to ensure that all the probes/genes only span coding sequences.

### Statistical analysis

Differential expression (genes) and genomic divergence (probes) were determined using the linear modeling analysis for microarrays implemented in the limma package^66^ with empirical Bayes adjustment to the variance. For both expression and sequence divergence records, three different linear models were fitted to the data and contrast matrices were created to identify (i) differences between ecotypes, localities and their interaction, (ii) differences between ecotypes within localities, and (iii) differences between localities for each ecotype. The resulting *p*-values were corrected for multiple tests using the binomial sequential goodness of fit procedure (SGoF)^67^ at α = 0.05.

For genes/probes showing significant differences between ecotype pairs in the three localities examined, we computed the *p*-value that the observed parallelism could be due to chance alone using both a randomization test^68^ and the algorithm developed by Derome *et al*.^69^ modified to include three localities (P. Duchesne, personal communication). Randomization tests were also used to estimate the random expectation of parallel and nonparallel changes, and of directional and nondirectional changes. For each randomization test, data were sorted 200,000 times and the corresponding outcome was obtained after multitest correction. An unpaired *t* test^70^ was used to test that intrapopulation variance differs between genes/probes showing directional versus nondirectional parallel changes. The program Blast2GO^71^ was used to identify which GO terms were significantly over-represented in those genes or probes showing significant differences for each analysis. General patterns of gene expression and sequence divergence were visualized with heat maps using R/Bioconductor.

## Results

We examined transcriptomes from pools including snails from the “crab” or “wave” ecotypes, and variation in the coding sequences of single snails. Snails were collected from three isolated, independently evolved population pairs of sympatric “crab” and “wave” ecotypes (Fig. 1) that previously showed a repeatable morphological divergence by parallel evolution^33,35,40^. Variation in expression and genomic sequence was determined for the same genes using a microarray specifically developed for *L. saxatilis*. After quality control of the hybridized arrays, we retained 22 out of 24 pools for gene expression, 69 out of 72 individuals for coding sequence divergence, and 17,431 genes.

Table 1 shows the proportion of genes displaying expression and genomic sequence differences between pairs of ecotypes for the three localities examined after using SGoF multitest correction (α = 0.05). Pairs of ecotypes living in the same site displayed significant differences in expression and genomic sequence, respectively, for up to 17.2 and 25.5% of all assayed genes. Transcriptomic differences were more prevalent than genomic differences in only one of the three localities assayed. The majority of divergent genes (93–94%) were divergent either for gene expression or genomic sequence, but not for both simultaneously. The number of differences between ecotype pairs varied among localities (P < 0.05; G test), and many of these differences (40.6–79% for gene expression; 68–71% for genomic divergence) occurred only in a single locality. Therefore, we tested whether differences between ecotype pairs frequently involved the same genes in the three localities (i.e. parallel changes). The observed numbers of genes with parallel changes in expression and genomic sequence were, respectively, 146 (0.8% of all assayed genes) and 216 (1.2%). Such levels of parallelism are highly unlikely just by chance (*p* < 10^−5^ for both expression and genomic data using a permutation test, or the algorithm by Derome *et al*.^69^), and therefore consistent with repeatable genetic differentiation by natural selection. Remarkably, as few as 15 genes displayed simultaneous parallel changes in expression and genomic sequence, representing 4% of all genes with parallel changes.

**Table 1:** Number of genes (percentage and absolute values) showing transcriptomic or genomic sequence differences between pairs of ecotypes in each single locality, and across the three localities simultaneously (parallel changes), after using SGoF multitest correction (α = 0.05).

Locality	Transcriptomics	Genomics	Transcriptomics and Genomics
Burela	17.2% (2998)	13% (2266)	1.9% (331)
Roncudo	6.4% (1115)	25.5% (4445)	2% (349)
Silleiro	5.9% (1028)	9.7% (1691)	1.1% (192)
N° of genes with parallel changes	0.8% (146)	1.2% (216)	0.08% (15)

The last column shows the genes that were divergent for gene expression and genomic sequence simultaneously. The total number of genes investigated is 17,431.

**Figure 2:**
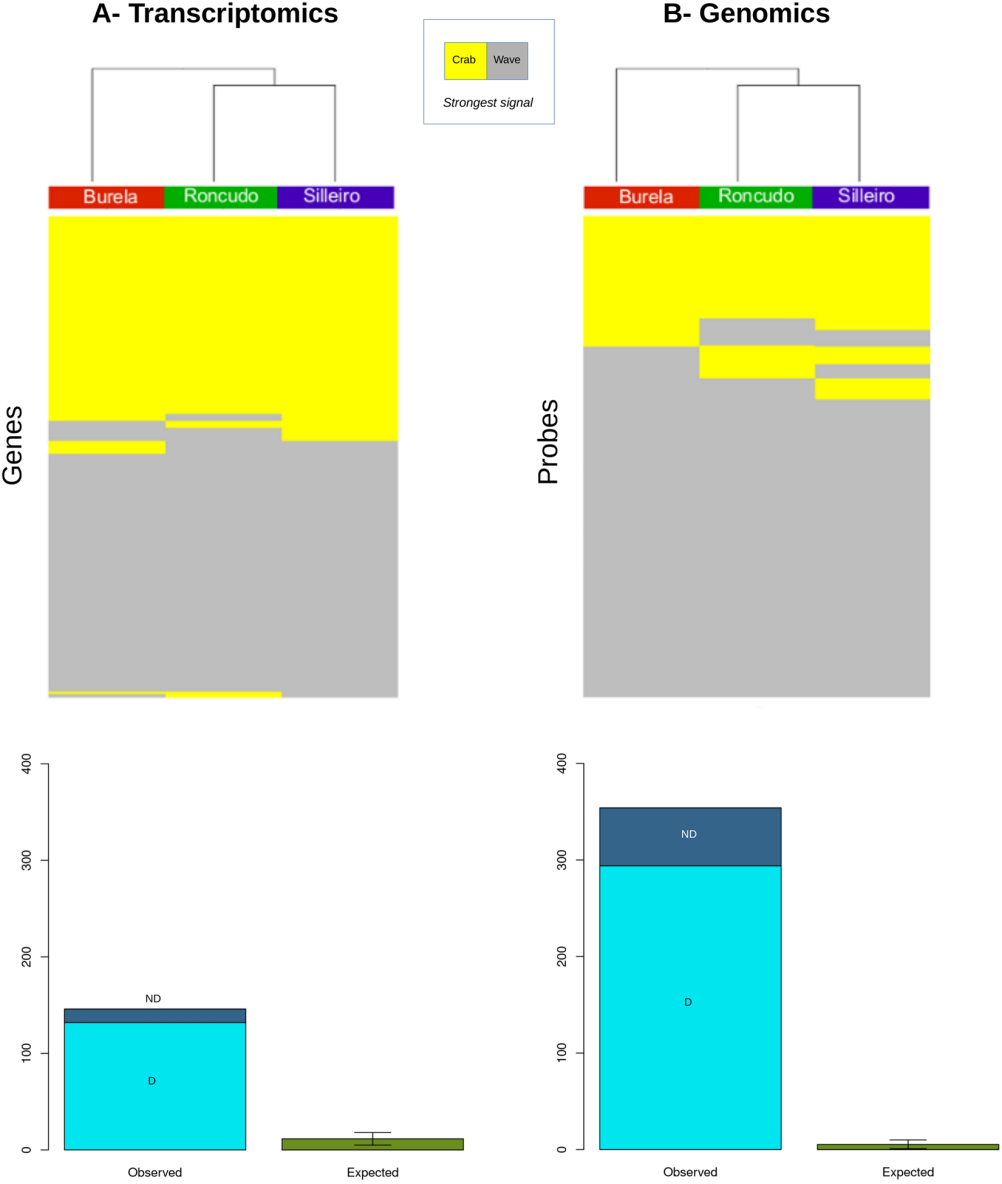
Directionality of observed parallel differences for (**A**) the transcriptomic and (**B**) genomic sequence scans. Top pannels: heat maps of hybridization signals across localities for the 146 genes (left) and 354 probes (right) that displayed significant parallel changes after multitest correction. Yellow indicates a greater hybridization signal for the “wave ecotype”, and grey a higher hybridization signal for the “crab ecotype”. Directional parallel changes appear as straight horizontal lines of one single color (yellow or grey). Nondirectional parallel changes display horizontal lines scattered of yellow and grey streches. Botton pannels: barplots indicating the observed number of directional (D) and nondirectional (ND) changes versus the random expectation, obtained using a randomization test (see methods). Vertical error lines indicate the 95% confidence intervals across all replicates of the randomization test.

We examined the directionality of observed parallel differences. To increase precision, genomic divergence is referred to in the subsequent analyses in terms of the 354 probes rather than the 216 genes that showed parallel changes (see methods). The majority of parallel differences between ecotype pairs were due to changes in the same direction (directional changes), whereas only a few were due to differences in opposite directions (non-directional changes) (Fig. 2). Specifically, up to 132 (90%) of all genes displaying parallel differences in expression showed directional changes (54% of which were up-regulated in the “crab ecotype”). Similarly, 294 (83%) of all probes with parallel variation in genomic sequence also showed directional changes (75% of which displayed a higher hybridization signal in “crab” than “wave” snails). The large over-representation of directional parallel differences for both expression and sequence divergence data is highly unlikely just by chance (each *p* < 0.0001), as determined by randomization tests resorting expression or genomic data sets (Fig. 2).

**Figure 3:**
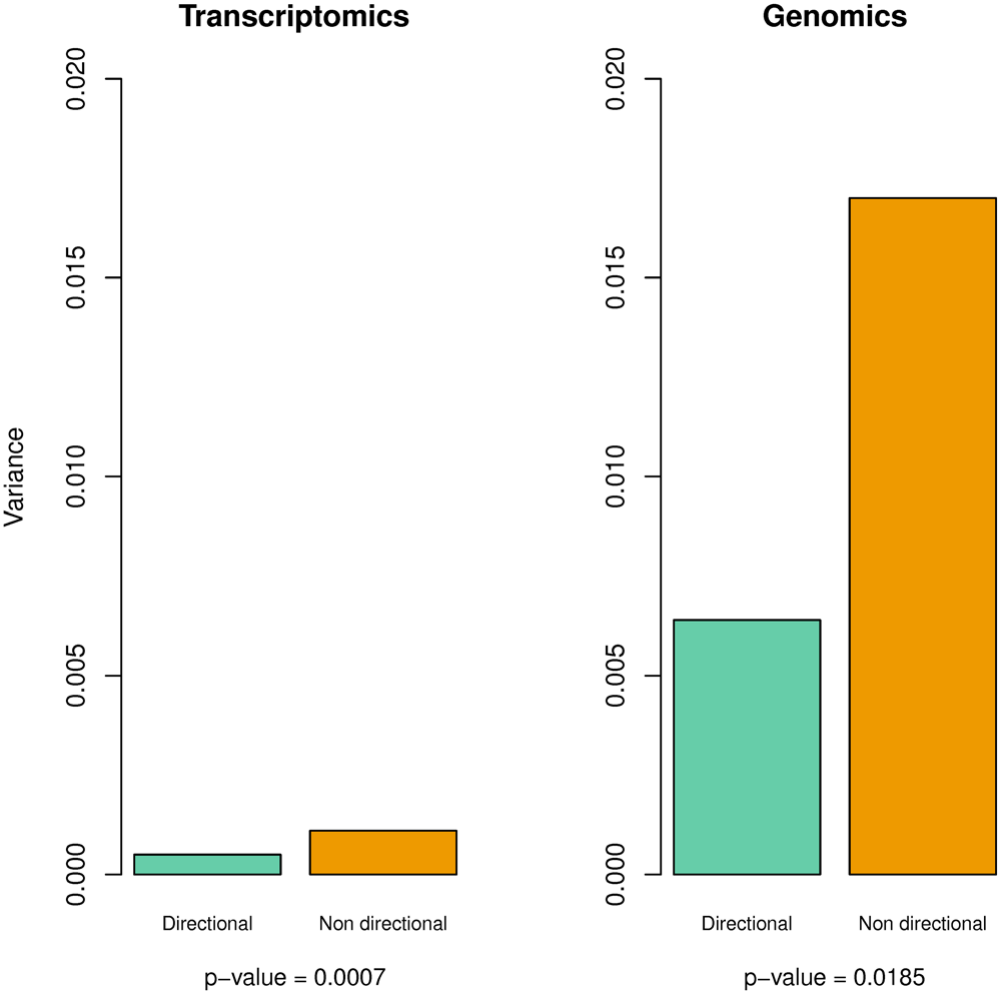
Mean intrapopulational variance in hybridization signal for directional parallel changes and nondirectional parallel changes. Variance for directional changes was significantly lower than that observed for nondirectional changes for both transcriptomic and genomic sequence scans.

We also determined whether the mean intrapopulation variance differs between genes/probes showing directional versus nondirectional parallel changes. We found that variance in expression and sequence divergence for directional changes was twice less than that observed for nondirectional changes (Fig. 3). These differences were statistically significant for both variation in expression (*p* = 0.0007) and genomic sequence (*p* = 0.0185) using a randomization test, and also using 2-tailed *t* tests (all *p* < 0.027).

**Figure 4:**
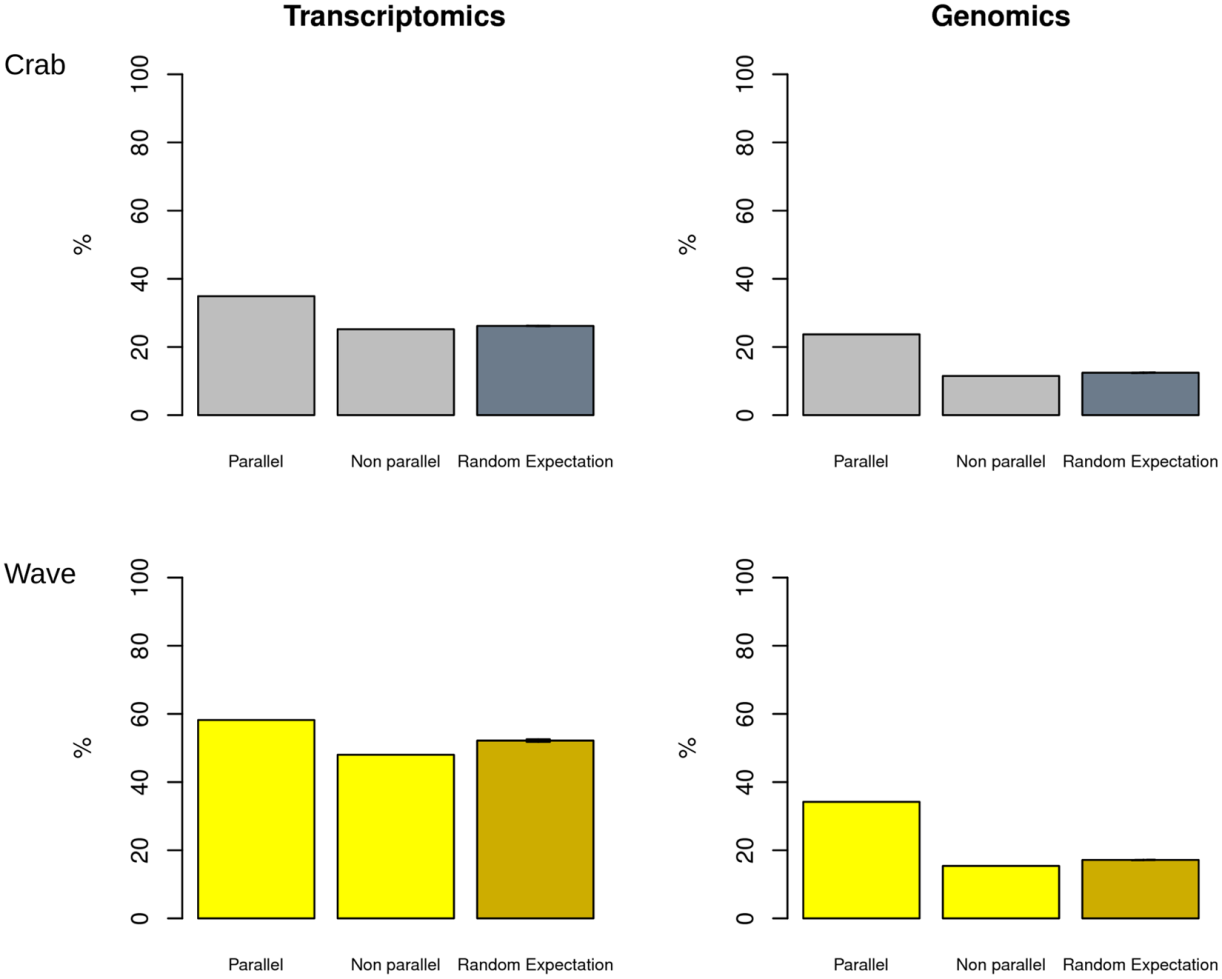
Geographical differentiation for genes (left) and probes (right) that displayed parallelism in gene expression or genomic sequence, and for those not showing parallelism. The y axis indicates the percentage of cases for which these genes/probes showed statistically significant differences among the three localities. Vertical error lines indicate the 95% confidence intervals for the random expectation (almost not appreciable in the figure), obtained resampling 1000 times at random the same number of genes/probes showing either parallel or nonparallel changes from the total set of genes/probes.

To further assess the nature of evolutionary forces underlying parallel variation, we determined which proportion of genes/probes showing parallel and nonparallel differences among ecotype pairs also showed a significant geographic differentiation among the three localities for the “crab” or “wave” ecotypes. We found that, independently of the ecotype considered, genes/probes with parallel changes showed more frequently geographic differentiation than genes/probes with nonparallel changes after SGoF multitest correction (α = 0.05), and that this pattern was more pronounced for genomic than transcriptomic differences (Fig. 4). The proportion of genes/probes with parallel changes that displayed geographic differentiation deviated more strongly (p < 0.001) from the random expectation than the proportion observed for nonparallel changes.

We used an enrichment analysis with BLAST2GO to test whether parallel differentiation is linked with specific functional groups. This analysis did not identify enriched gene/probe sets after correction for multiple testing when the whole data set or only intra-site GO enrichment tests were considered. Nevertheless, some genes/probes with the most extreme parallel directional changes in hybridization signal included annotations expected to be involved in adaptation. For example, the Dermatopontin 2 (for gene expression profiling) and the Keratin-associated protein 4–3 (for sequence divergence profiling) are involved, respectively, in the formation of the shell^72^ and the operculum^73^, key features defining differences between ecotype pairs (Supplementary Tables S1 and S2). We also tested whether the differences between ecotype pairs that are unique to each locality are linked with specific functional groups. For this analysis, significant enrichment GO terms were observed only for gene expression profiling after correction for multiple testing. We observed an important enrichment in energetic metabolism GO terms for Burela, but almost no GO terms were shared among pairs of localities, and none between the three localities simultaneously, either for the categories of molecular function, biological process, or cellular component (Supplementary Figs S1 and S2).

## Discussion

### Parallelism in gene expression and coding sequences

We report evidence that parallel differences in expression and sequence divergence of a limited set of genes underlay the repeated phenotypic divergence of replicate pairs of *L. saxatilis* ecotypes. The observed numbers of parallel differences in gene expression and sequence divergence largely exceeded the random expectation. Several results suggest that adaptive selection played a role, direct or indirect, in the process of molecular divergence among ecotypes.

We expect that genes repeatedly recruited by strong natural selection would show striking habitat-associated differences^74^, would display less variation than those under weaker selection^69^, and would show a higher geographical differentiation^75^. Overall, our results fit these expectations and are consistent with a scenario in which the same subset of genes, or regulatory regions, were repeatedly recruited by natural selection in populations adapted to similar habitats. Parallel changes in hybridization signal were nearly restricted to directional changes, denoting a repeated and significant habitat-association among independently evolving populations of similar phenotype that cannot be explained by chance. Therefore, directional parallel changes showed a lower intrapopulation variance than nondirectional parallel changes, as expected from a stronger impact of selection in the former^69,76^. Also, the distinctive higher geographic differentiation in expression and coding sequence for genes displaying parallelism did not match the random expectation. This in turn suggest that geographic differentiation for genes showing parallelism is determined by the joint action of divergent selection and stochastic forces, whereas geographic differentiation at nonparallel genes is mostly driven by stochastic forces. Last, we examined the function of genes with parallel divergence. Although annotation was very incomplete due to the poor representation of mollusk sequences in public databases^77^, some of the genes that could be annotated exhibited functions related with well know adaptive phenotypic characters, such as the formation of the snail shell and the operculum.

Despite the observed parallelism, the majority of differences in gene expression and coding sequence were not shared among localities. Low sharing of divergent genes contrasts with the expectation of high gene reuse for the parallel evolution of individual phenotypic traits among closely related taxa and populations^9^. Several non-mutually exclusive factors may account for this discrepancy. First, we might have underestimated the parallelism existing in natural populations. For example, parallelism owing to low diverged alleles, or to alleles equally diverged from the reference but carrying mutations at different sequence positions, could remain somewhat undetected using microarrays. Sequence mismatches due to sequence polymorphisms could also affect the ability to detect parallelism in gene expression. Therefore, the number of genes showing parallelism in our study should be viewed as conservative. Yet, the impact of these challenges on our patterns of parallelism seems to be modest since we detected many differences between ecotype pairs of a very recent origin within each locality, and still only a minor fraction of these differences were repeatable among localities. Moreover, expression measurements in different species did not reveal a consistent variation in signal intensity due to sequence mismatches^24,78^, since the expression of each gene is calculated as the average intensity for each probe set. Second, if divergent traits in *Littorina* (e.g. shell size and shell shape) are highly polygenic, then they may show greater genetic redundancy than traits determined by a single gene or molecular pathway. As such, changes in different pathways of a complex polygenic trait could lead to similar phenotypes and show less repeatable genetic signatures of adaptation^3,22^. Third, patterns of parallel evolution could be more common at higher levels of biological organization^79^. Thus, sharing of physiological processes, biochemical pathways, or organismal functions may therefore be more prevalent than observed at the gene or regulatory level^7,80–83^. Last, a number of biases could have inflated the very high expectation of gene reuse, such as publication bias against non-sharing genetic patterns, or an emphasis on genes of large effect that may not be illustrative of the true spectrum of phenotypes^3,9,84^.

Other studies in a number of different organisms have similarly demonstrated little sharing of sequence divergence^10,13,85,86^ and gene expression patterns^69,87^ linked to recent parallel evolution. Our findings are consistent with recent genome scan studies in *Littorina* indicating a low sharing of genomic divergence among ecotypes that arose in parallel in different parts of Europe but also, as shown in Sweden, in geographically close localities^42,44^. Moreover, parallelism between ecotype pairs mostly involved genomic regions under strong selection^42,43^, thus supporting our hypothesis that genes showing shared genomic and expression divergence are likely targeted by natural selection.

About 10% of sequence differences in the *Littorina* array are expected to be copy number variants^58^. Thus, processes such as duplication and subsequent neofunctionalization might also play a role in the divergence among ecotypes^4,22,88^. Our results show that the *Littorina* microarray is able to detect more sequence differences among ecotype pairs than reported in a previous study using this same microarray^58^. Several reasons explain this gain in power. In the former study, a reference sample was not used and data was not filtered, thus increasing the inter-array variance due to technical noise effects^89^. Also, a probe-based analysis was not used to assess sequence differences. Instead, the relative hybridization signal for each gene represented on the array was calculated as the *average* intensity for *each probe set*. Thus, any mismatch signal resulting from a target DNA polymorphism affecting one single probe would be averaged with the remaining gene probes and therefore would be difficult to detect. In addition, sequence comparisons between ecotype pairs within a single locality were based on only four individuals from each ecotype. To obtain more power, in the present study the sample size was increased to 12 “crab” and 12 “wave” individuals per locality (72 individuals in total versus 8 in the former study for Galician snails). Finally, we used the limma package^66^ with empirical Bayes adjustement to the variance, that allows an improved estimation of variance respect to the conventional ANOVA tests previously used.

The chances of successfully capturing adaptive loci are greater when targeting functionally important regions. In this study, we simultaneously screened patterns of expression and sequence variation for the coding fraction of the genome. Thus, although some of the genes displaying parallel evolution could be false positives, the likelihood of identifying genes directly or indirectly (through linked selection) targeted by selection may be substantial. Remarkably, a large number of divergence events occurred in a single ecotype pair. An unknown proportion of this non-shared divergence could have resulted from stochastic processes, adaptive changes, or a combination of these factors. Overall, our results suggest that the genomic architecture underlying parallel phenotypic divergence probably followed a complex evolutionary path, affecting multiple loci in a mosaic pattern of both repeatable and idiosyncratic divergence, and where the repeated element involved many regions affected by natural selection.

### Divergence in gene expression is decoupled from divergence in coding sequence

Our results showed that patterns of differentiation in gene expression and coding sequence were markedly dissimilar. The majority of divergent genes were divergent either for gene expression or genomic sequence, but not for both simultaneously. Remarkably, as few as 15 genes displayed simultaneous parallel changes in expression and genomic divergence, representing 4% of all genes with parallel changes. Such result is consistent with the hypothesis that expression and gene sequence differences underlying phenotypic divergence could, at least to a certain extent, be considered decoupled processes^31^.

One concern is that the comparison between expression and sequence variation could have been partly affected by misleading expression measurements resulting from sequence mismatches between the samples used for expression analysis and the reference upon which the array was designed. However, sequence mismatches cannot account for the dissimilarity in patterns of differentiation, since such mismatches should also be present in the samples used for sequence differentiation and would generate a correlated signal between gene expression and sequence divergence^90^. It might be also possible that our genome scan was not sensitive enough to pick up all the genes carrying a single nucleotide variant difference. However, this lack of sensitivity should equally affect the coding regions of genes displaying either expression or no expression differences, and thus cannot explain the dissimilarity. Also, for gene duplications where both genes are retained, similar patterns of differentiation are expected for gene expression and gene sequence if both diverge at clock-like rates^91^. It is also unlikely that power differences between expression and sequence divergence studies can account for the dissimilarity in patterns of differentiation, as they should lead to consistently larger differences between ecotype pairs for one such level (expression or sequence divergence) in the three localities examined and, therefore, genes with significant differences in the less powerful study should also display concordant significant differences for the most powerful one. These patterns are not observed in our data (Table 1). These considerations further support that, independently of the source of variation or error considered, gene expression and coding sequences appear to evolve differently as ecotypes repeatedly adapt to complex ecological gradients.

The decoupling between gene expression and coding sequence differentiation is consistent with the existence of trans-regulation factors driving gene expression evolution, but also with the evolutionary decoupling of cis-regulatory regions and coding sequences. For example, in *Drosophila melanogaster*, gene expression differences among distinct strains are correlated with 5 prime sequences but not with coding sequences, thus supporting that differentiation at cis-regulatory regions is decoupled from differentiation at coding regions^92^. Our results are in line with what was observed among closely related ecotypes of lake whitefish^20^, rainwater killifish^93^, and woody sunflower^29^, where differentiation of gene expression and coding sequences were also decoupled. However, what distinguishes our study from these previous ones is that we focus on genes displaying parallel evolution across similar environmental gradients. As such, the genes we identify are more likely to underlie variability related with traits implied in a relevant adaptive response.

Functional interpretations of the decoupling between gene expression and sequence divergence should be taken cautiously, as array data do not allow to tell apart effects due to nonsynonymous mutations that alter the amino acid sequence from those due to synonymous mutations that do not affect the amino acid composition. Yet, even if most of changes occurred at synonymous sites, it would be needed to explain why in our data differentially expressed genes do not show such changes. This would point to the existence, even for synonymous sites, of selective constraints slowing down the evolution of coding sequences for genes displaying parallel changes in expression. Indeed, evidence exists indicating that synonymous sites appear to evolve slower than expected under neutrality in a way apparently consistent with weak selection in organisms as diverse as insects, yeast, worms, chicken or mammals^94–98^. For example, in *D. melanogaster*, 22% of four-fold synonymous sites are evolving under strong constraints, and genes with such constrained sites tend to be especially relevant, highly expressed, and often involved in developmental networks^99^. Therefore, our results may point to the possibility of some division of tasks underlying coding and regulatory regions, as previously hypothesized^100^.

Genome-wide data on expression variation versus sequence divergence are uncommon. Thus, this study provides a rare opportunity to determine the relative contribution of expression and coding changes underlying parallel phenotypic evolution. Our results suggest that both coding and expression changes contribute to parallel divergence among pairs of ecotypes. The relative contribution of expression and sequence changes varied among localities, but there was not an overall preeminent role of expression over coding sequence differences across all localities. Our results differ from other studies in three-spined sticklebacks providing a major role to gene expression variation (up to 83% of all differences) over coding sequence variation in the evolution of parallel phenotypic divergence^16^. This suggests that differences in life history features and the number, location and interactions among genes and regulatory regions, may generate very diverse outcomes in the molecular fingerprint underlying phenotypic adaptation^23^.

## Electronic supplementary material

Supplementary information

## Data Availability

The expression and genomic divergence dataset is available in the NCBI gene expression Omnibus under the accessions GSE120697 and GSE120698 respectively.
