# Supplementary material for: Population genomics of parallel evolution in gene expression and gene sequence during ecological adaptation
Source: Sci Rep. 2018 Nov 1;8:16147. doi: 10.1038/s41598-018-33897-8 (PMC6212547; doi:10.1038/s41598-018-33897-8)

Supplementary Information

Population genomics of parallel evolution in gene expression and gene sequence during ecological adaptation

María José Rivas[1], María Saura[1], Andrés Pérez-Figueroa[1], Marina Panova[2], Tomas Johansson[3], Carl André[2], Armando Caballero[1], Emilio Rolán-Alvarez[1], Kerstin Johannesson[2], Humberto Quesada[1*]

[1]*Departamento de Bioquímica, Genética e Inmunología, Universidad de Vigo, 36310 Vigo, Spain*

[2]*Department of Marine Sciences, Tjärnö, University of Gothenburg, SE-452 96 Strömstad, Sweden*

[3]*Department of Biology, University of Lund, SE-223 62 Lund, Sweden*

***Correspondence:** Humberto Quesada. E-mail: hquesada@uvigo.es

Supplementary Table S1. Blastn results for genes with the most extreme parallel directional changes in gene expression between pairs of ecotypes.

Gene microarray code[1]	Accession[2]	Annotation	FC[3]
c2540	B5LYM3	Dermatopontin 2	-4.17
c4038	Q01528	Hemagglutinin/amebocyte aggregation factor	-4.12
c3616	D2VG42	Predicted protein	2.66
c22794	G3US88	Uncharacterized protein	2.62
C6561	P08548	LINE-1 reverse transcriptase homolog	-2.47
c921	A7RTH9	Predicted protein	2.43
c20264	H2L5T7	Uncharacterized protein	-2.39
c8805	D2A572	Putative uncharacterized protein GLEAN_15234	2.30

[1] Identification code within the array. [2] Accession code in *Uniprot* database (www.uniprot.org). [3] Relative change in gene expression *(fold change).* Positive values of *FC* mean an over-expression in the "wave ecotype", while negative values of *FC* mean over-expression in the "crab ecotype". An *e-value* < 9E-18 was used for the Blastn search.

Supplementary Table S2. Blastn results for genes with the most extreme parallel directional changes in genomic sequence hybridization signal between pairs of ecotypes.

Gene microarray code[1]	Accession[2]	Annotation	FC[3]
s26531	F7EBV1	Uncharacterized protein	2.49
C25426	C3ZWI9	Putative uncharacterized protein	1.82
c9191	B4JMZ3	GH24718	1.67
c354	F7EBV1	Uncharacterized protein	1.63
c9430	C3XYW3	Putative uncharacterized protein	1.61
c2581	C3XYW3	Putative uncharacterized protein	1.58
c19038	E7D1V6	Putative uncharacterized protein	1.55
c9430	C3ZWI9	Putative uncharacterized protein	1.55
c470	G3HU38	Keratin-associated protein 4-3	-1.51
c26438	C3ZWI9	Putative uncharacterized protein	1.49
c2581	C3XYW3	Putative uncharacterized protein	1.48
c2621	P30682	Guanine nucleotide-binding protein G(i) subunit alpha	-1.41
c1400	A7SS32	Predicted protein	-1.37
c834	B9DQU4	SCO-spondin	-1.37

[1] Identification code within the array. [2] Accession code in *Uniprot* database (www.uniprot.org). [3] Relative change in in hybridization signal *(fold change)* for the gene probe displaying the largest difference between pairs of ecotypes. Positive values of *FC* mean a higher hybridization signal in the "wave ecotype", while negative values of *FC* mean a higher hybridization signal in the "crab ecotype". An *e-value* < 9E-18 was used for the Blastn search.

Supplementary Figure S1. Venn Diagrams showing the overlap between GO terms over- and under-represented for locality-specific expression differences. For this analysis, the categories for the GO terms included biological process, molecular function, and cellular component, each with a FDR < 0.05.

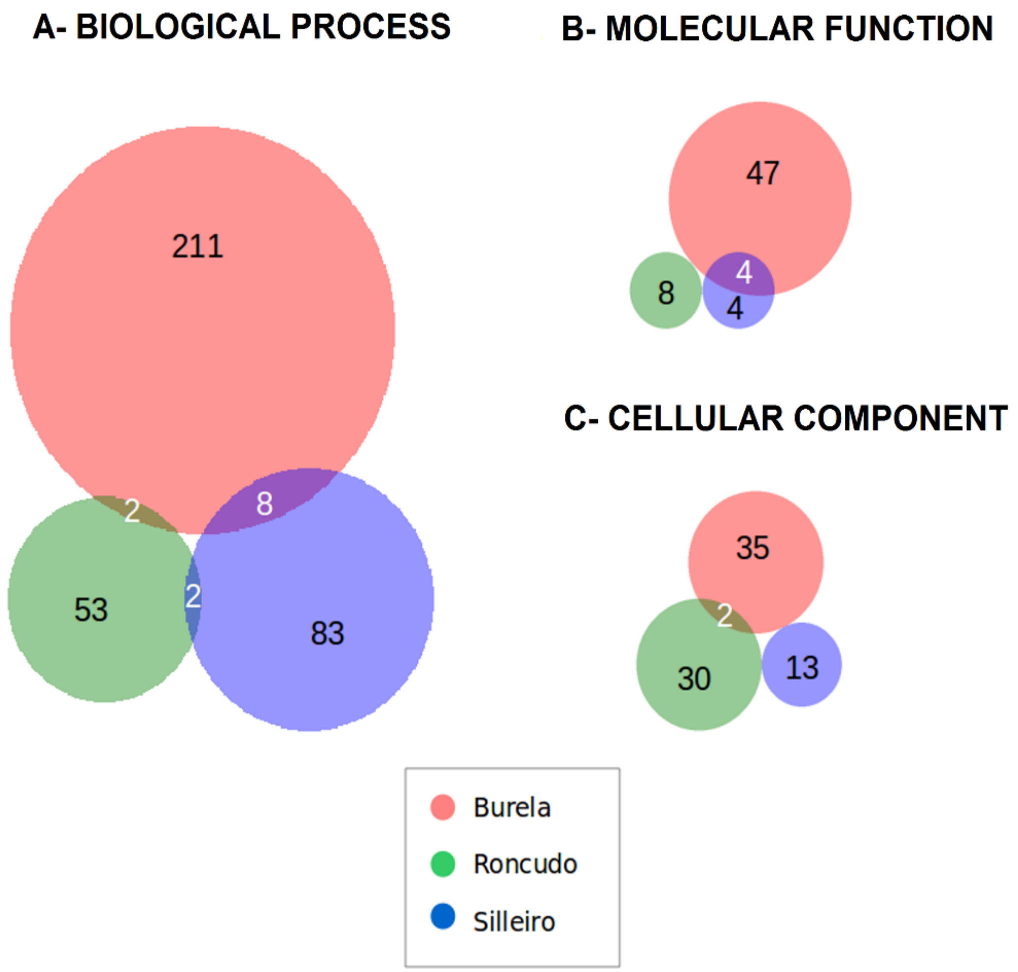

Supplementary Figure S2. Bar graphs show the enriched GO terms and the number of probes concerned for the reference group (all annotated genes) and the tested group (genes with expression differences specific of locality). A longer red than blue bar indicates that the corresponding GO term is over-represented. Alternatively, a longer blue than red bar indicates that the GO term is under-represented. For this analysis, the categories for the GO terms included biological process, molecular function, and cellular component, each with a FDR < 0.05. Note that not all the GO terms have been spelled out.

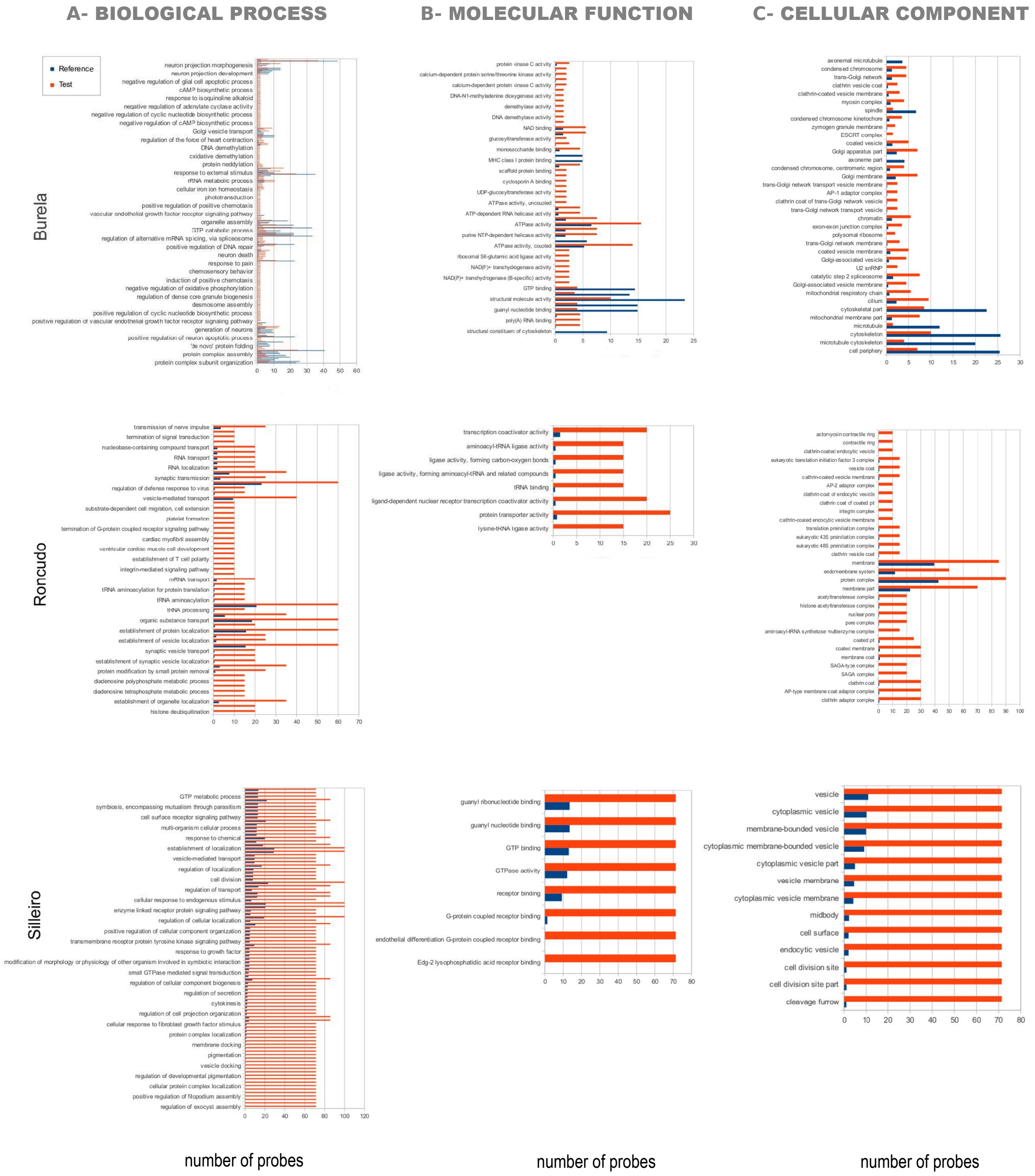

Supplement: Supplementary file 1 — Supplementary information [file 41598_2018_33897_MOESM1_ESM.pdf]
